# High penetrance, recurrent attacks and thrombus formation in a family with hereditary coproporphyria

**DOI:** 10.1002/jmd2.12281

**Published:** 2022-03-18

**Authors:** Cindy Towns, Sobana Balakrishnan, Chris Florkowski, Andrew Davies, Elaine Barrington‐Ward

**Affiliations:** ^1^ Department of General Medicine Wellington Regional Hospital Wellington New Zealand; ^2^ Department of Medicine, Wellington School of Medicine University of Otago Wellington New Zealand; ^3^ Clinical Biochemistry Unit Canterbury Health Laboratories Christchurch New Zealand

**Keywords:** encephalopathy, hepatic, ketamine, opioid‐related disorders, porphyrias, status epilepticus, thromboembolism

## Abstract

Hereditary coproporphyria (HCP) is the rarest of the autosomal dominant acute porphyrias with an estimated incidence of 0.02 per 10 million per year. HCP has been considered to be mild in presentation compared with the more common acute intermittent porphyria although there is limited information comparing the subtypes. Penetrance in the acute porphyrias is low with 90% of patients with a mutation never exhibiting symptoms. We present seven members from a family with HCP with a novel mutation in whom penetrance and severity are high. In addition, they appear to have a high rate of veno‐thromboembolism. Penetrance is confirmed at 57% but is suspected to be 71%. The first patient experienced life‐threatening complications, four of the seven have had recurrent attacks and the development of opioid dependence has complicated management. The case series documents the impact of a new mRNA interference molecule givosiran as well as a plan for embryo selection which is not commonly used in porphyria. The use of ketamine for the treatment of acute attacks is also documented for the first time in the porphyria literature. The use of international registries would aid the characterisation and management of this very rare disease.

**Synopsis**

Traditionally believed to have low penetrance and possibly milder presentations, this hereditary coproporphyria case series describes a highly penetrant cohort suffering recurrent attacks and severe complications including status epilepticus and posterior reversible encephalopathy as well as opioid dependence; the role of novel management strategies such as ketamine, givosiran and embryo selection are highlighted.

## 1 INTRODUCTION

The porphyrias are rare disorders of haem synthesis. The autosomal dominant acute porphyrias include acute intermittent porphyria (AIP), variegate porphyria (VP) and hereditary coproporphyria (HCP). Penetrance is low with few suffering recurrent attacks.^1^ Little is known about HCP, the rarest subtype with an estimated incidence of 0.02 per year per 10 million.^2^ Acute porphyria is not known to be associated with venous‐thromboembolism (VTE).

We present seven cases of HCP from a single family with a novel mutation who have exhibited an unusually high penetrance and high rates of severe, recurrent attacks. There is also a high incidence of VTE.

## 2 CASE PRESENTATIONS

### 2.1 Case 1 (III:1)

Case 1 is a 21‐year‐old woman with a background of a single seizure in adolescence. She had an intrauterine device (MIRENA) in situ for several years. Her initial attack was life‐threatening with severe abdominal pain, status epilepticus and posterior reversible encephalopathy.^3^ The precipitant was viral gastroenteritis with dehydration.

Testing confirmed a raised urinary porphobilinogen (PBG) of 12.4 μmol/mol creatinine (normal <1.5). Urinary porphyrin/creatinine ratio was 673 nmol/mmol (normal <35), faecal porphyrins were 2430 μmol/kg dry weight (normal <200) and metabolite profiles (raised CIII:CI ratio) confirmed acute HCP. Genetic testing revealed a novel missense variant in the coproporphyrinogen oxidase (CPOX) gene c.863 T > G (p.Leu288Trp). Intravenous (IV) haem arginate was administered to treat her acute attack. She developed a deep vein thrombus (DVT) at the infusion site in the antecubital fossa and was anti‐coagulated with warfarin for 3 months.

She has had four subsequent admissions with acute attacks. Community testing shows several episodes of raised PBG in the context of abdominal pain. She admits to avoiding hospital due to anxiety related to her ICU admission. The MIRENA remains in situ.

Following VTE in her sister and cousin, a thrombophilia screen was conducted. She had normal tests for anti‐nuclear antibodies (ANA), antithrombin III, protein C and S, anticardiolipin, d‐dimer, anti‐ds‐DNA, lupus anticoagulant, factor V Leiden and the prothrombin gene mutation (G20210A).

**FIGURE 1:**
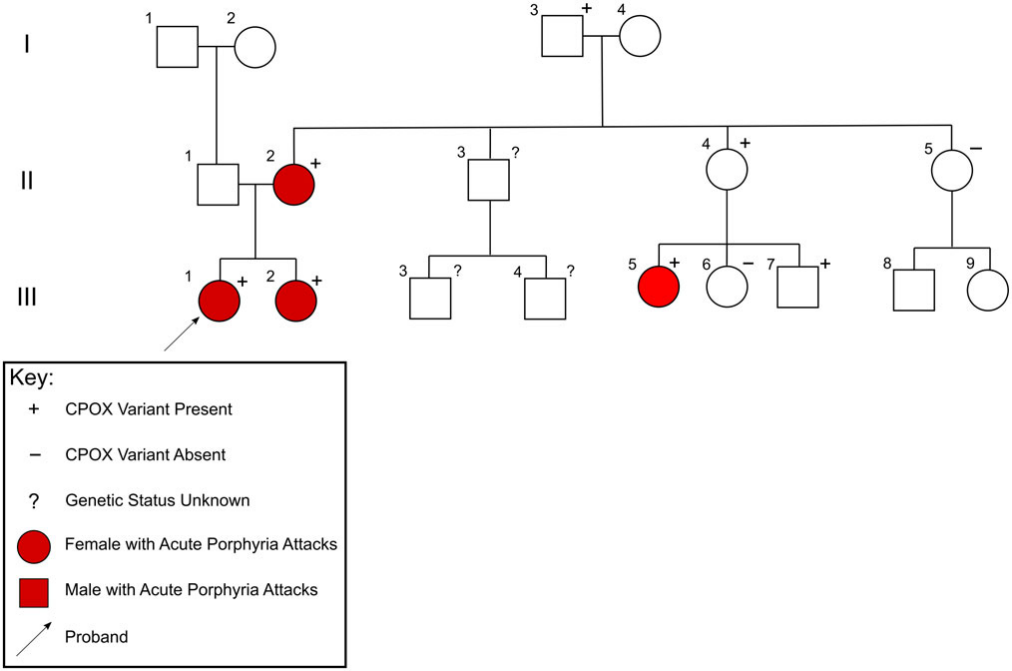
Pedigree showing distribution of CPOX variant gene c.863 T > G (p.Leu288Trp). Arrow depicts proband

Cascade genetic testing has revealed six other family members who carry the CPOX mutation (Figure 1). It should be noted that some family members (II:3, III:3 and III:4) have not accessed the available genetic testing.

### 2.2 Case 2 (III:2)

Case 2 is a 23‐year‐old woman and the sister of case 1. She suffers depression and anxiety and had a previous laparoscopy for dysmenorrhoea with right ovarian cystectomy. Her first attack was characterised by severe abdominal and proximal leg pain. She had a raised urinary PBG of 3.1 μmol/mol creatinine (normal <1.5). and a raised urine porphyrin: creatinine ratio of 186 (reference range: 0–35). She received IV haem arginate via a central line. HCP was confirmed with elevated faecal porphyrins (raised CIII:CI ratio), and genetic testing demonstrated the familial mutation.

She has had 43 admissions with numerous confirmed attacks. She also suffers pain flares without raised PBG. Her treatment has been complicated by two VTE. The first was in the right cephalic vein superior to a peripherally inserted central catheter (PICC) for which she received enoxaparin. The PICC line was removed, and a port‐A‐Cath was placed. She then developed a new non‐occlusive thrombus around the port‐A‐Cath in the mid right subclavian vein despite enoxaparin. She was switched to dabigatran for 6 months.

The oral contraceptive pill was trialled (AVA 20 – levonorgestrel and ethinylestradiol) with two active packs taken together to reduce fluctuations in progesterone. This did not reduce the frequency of attacks. A MIRENA was then placed which appeared to increase the frequency of attacks despite case 1 remaining well with her Mirena.

High doses of opioids have been required. Non‐steroidal anti‐inflammatory drugs (NSAIDs), gabapentin and tricyclic agents have had little benefit. Ketamine infusions have been trialled with a decrease in pain severity. However, akathisia developed during one admission. Although deemed multifactorial ketamine was discontinued as a precaution.

In 2018, she was enrolled in a clinical trial with the novel RNAi molecule givosiran. Hospital admissions and pain severity have significantly reduced. Homocysteine levels were found to be high during the trial (20 μmol/L; normal range: 5–15). She has subsequently been prescribed a multivitamin containing folic acid, vitamin B6 and vitamin B12.

### 2.3 Case 3 (III:5)

Case 3 is a 22‐year‐old female cousin of cases 1 and 2. She has a history of depression, body dysphoria and low body mass index (BMI). She was identified as carrying the mutation on genetic testing and faecal metabolite profile confirmed HCP. She has had 19 admissions with acute attacks and pain flares. Acute attacks with raised PBG were treated with haem arginate via central or PICC line. She also developed recurrent thrombi. She developed a right cephalic vein occlusive thrombus in the arm used for haem arginate administration with an associated *Staphylococcus aureus* bacteraemia and also thrombi in the left internal jugular vein and a superficial vein in the left elbow (not used for infusion). Management has been complicated by opioid dependence.^4^

She remains on dabigatran. Thrombophilia screening was negative for lupus, beta2 glycoprotein antibodies and paroxysmal nocturnal haemoglobinuria. She has a normal lactate dehydrogenase (LDH), homocysteine, immunoglobulins and CD4/CD8. She is planning a family. She has requested in vitro *fertilisation* to enable pre‐implantation diagnosis to avoid passing on the CPOX mutation.

### 2.4 Case 4 (II:2)

Case 4 is a 44‐year‐old woman and the mother of cases 1 and 2. She has a history of anxiety, depression and abdominal pain with exploratory laparoscopy. Genetic testing confirmed the mutation and she has elevated faecal porphyrins with an HCP profile. She has had one confirmed attack with abdominal pain and increased PBG excretion. She has not represented but has raised PBGs in the community. There is no history of VTE.

### 2.5 Case 5 (II:4)

Case 5 is a 55‐year‐old postmenopausal woman and the mother of case 3 and sister of case 4. She suffers from anxiety and depression and has seronegative rheumatoid arthritis. Genetic testing has confirmed the mutation. She has raised faecal porphyrins with a metabolite profile consistent with HCP. Although she has no documented attacks, she has a history of severe abdominal pain which resolved following menopause. There is no history of VTE.

### 2.6 Case 6 (I:3)

Case 6 is an 83‐year‐old gentleman and the father of cases 4 and 5. Genetic testing revealed the mutation. His urinary porphyrins and faecal porphyrins were normal. He has no history consistent with acute attacks hence he appears to be latent.

### 2.7 Case 7 (III:7)

Case 7 is a 31‐year‐old man and the brother to case 3. He has no history consistent with acute attacks. Urinary porphyrins were within the normal range but faecal porphyrins were mildly raised with an HCP profile. Genetic testing confirmed the familial CPOX mutation. He too appears to be latent.

## 3 DISCUSSION

This cohort is unusual for several reasons. First, the penetrance is very high. Penetrance in the acute porphyrias is low with 90% of affected individuals never experiencing an attack.^1^ Data on HCP are limited given the rarity of the condition but one family had only one of the 14 members exhibit symptoms.^5^ A confirmed penetrance of 57% (suspected to be 71%) in this cohort is unusually high (NB: II:3, III:3 and III:4 were not included in this calculation given that their genetic status with regard to the CPOX variant is unknown). Given that all four women with confirmed attacks are of reproductive age and that both males are latent, we speculate that penetrance for this mutation is particularly sensitive to hormonal fluctuations. This is consistent with case 5 who stopped suffering abdominal pain following menopause. Family studies in AIP have demonstrated that disease penetrance is higher in clinically affected families when compared with the general population.^6^, ^7^ It is possible that similar modulation, by shared genetic modifiers or common environmental factors, also occurs in the rarer HCP.

Second, this family is suffering an unusually high rate of recurrent attacks. Recurrent attacks are defined as four or more needing hospital admission in 1 year.^1^ Of those who experience an acute attack less than 10% develop recurrent attacks.^5^ Severe recurrent acute attacks affect 3%–5% of newly diagnosed symptomatic patients and are more common in AIP and in females.^2^ Cases 2 and 3 meet the criteria and it is possible that case 1 does too although she manages largely in the community. The fluctuations and reports of abdominal pain in case 4 suggest that she too suffers recurrent attacks. Given that ‘hospital admission’ is a subjective threshold (with reliance on patient and physician factors as well as access to care), it is plausible that four of the seven suffer recurrent attacks. If hospitalisation were not required for definition, then recurrent attacks cannot be excluded in case 5. Regardless of definition, the proportion suffering recurrent attacks in this cohort appears to be higher than predicted. This stands in contrast to a previous study of 108 patients with acute porphyria in which disease manifestations were less frequent and less severe in HCP and VP when compared to the more common AIP.^8^ A later review has also stated that attacks are much less frequent in HCP and VP than in AIP.^9^

Finally, the incidence of VTE is high. Three of the six developed thrombus and two developed recurrent thrombi requiring extended anticoagulation. It is possible that haem arginate contributed to the first thrombus (case 1) due to infusion through a peripheral vein. However, subsequently all patients were infused centrally after dilution in albumin to increase solubility and lower the risk of vein injury.^10^, ^11^ Coagulopathies reported with other haem preparations do not develop with stabilised haemin with arginine so the high occurrence cannot be attributed to this alone.^10^ Furthermore, a new thrombus formed while case 2 was on enoxaparin and in case 3, a DVT developed in the non‐infusion arm. Inherited thrombophilia testing is unremarkable and cases 4 and 5 had pregnancies uncomplicated by VTE. It is possible that there is a weak hereditary predisposition to VTE in this family but provocation from IV catheters, central lines, dehydration and acute illness are likely to be contributing significantly.

Although retrospective studies have at times been conflicting, two large cohorts have shown an association between high homocysteine levels and VTE.^12^ Other recent research has demonstrated higher than normal levels in symptomatic AIP patients and those receiving haemin or givosiran.^13^ Homocysteine is not collected as part of routine thrombophilia screening at Wellington Hospital and although elevated in case 2, the level was only measured following multiple haem infusions and the prescription of givosiran; hence, it is unclear whether the level was raised prior to the development of VTE or the administration of these agents.

In summary, this HCP cohort has unusually high penetrance and recurrent attacks compared with the literature. This is speculated to be due to increased susceptibility to hormonal factors. In addition, this cohort may have an elevated risk for VTE but this remains speculative. Given that HCP is a very rare disease, international registries may aid in our understanding of this condition.

## CONFLICT OF INTEREST

All the authors declare that they have no conflict of interest.

## AUTHOR CONTRIBUTIONS

Cindy Towns conceptualised the study, contributed to the literature review, conducted the clinical analysis, supervised Sobana Balakrishnan wrote and reviewed the draft manuscripts. Sobana Balakrishnan conducted the literature review, collected the clinical data, contributed to the clinical analysis and wrote the original draft. Andrew Davies and Elaine Barrington‐Ward contributed to the clinical analysis and management, supervised Sobana Balakrishnan and reviewed and edited draft manuscripts. Chris Florkowski conducted the laboratory analysis and laboratory data collection, provided supervision of laboratory staff and reviewed and edited draft manuscripts. Cindy Towns and Chris Florkowski verified the data. The authors have reviewed the final version to be published and are responsible for the accuracy and integrity of the manuscript.

## ETHICS STATEMENT

All patients gave verbal and written consent. No ethics approval was required.

## Data Availability

Data sharing not applicable to this article as no datasets were generated or analysed during the current study.
